# Hydroxyapatite/TiO_2_ Nanomaterial with Defined Microstructural and Good Antimicrobial Properties

**DOI:** 10.3390/antibiotics11050592

**Published:** 2022-04-28

**Authors:** Miljana Mirković, Suzana Filipović, Ana Kalijadis, Pavle Mašković, Jelena Mašković, Branislav Vlahović, Vladimir Pavlović

**Affiliations:** 1 Department of Materials, University of Belgrade, “Vinča” Institute of Nuclear Sciences—National Institute of the Republic of Serbia, 11000 Belgrade, Serbia; anaudovicic@vin.bg.ac.rs; 2 Institute of Technical Sciences of SASA, KnezMihailova 35/IV, 11000 Belgrade, Serbia; suzana.filipovic@itn.sanu.ac.rs; 3 Faculty of Agronomy in Čačak, University of Kragujevac, Cara Dušana 34, 32012 Čačak, Serbia; pavlem@kg.ac.rs (P.M.); jelenav@kg.ac.rs (J.M.); 4 Department of Mathematics and Physics, North Carolina Central University, Durham, NC 27707, USA; vlahovic@nccu.edu; 5 NASA University Research Center for Aerospace Device Research and Education and NSF Center of Research Excellence in Science and Technology Computational Center for Fundamental and Applied Science and Education, Durham, NC 27707, USA; 6 Department for Physics and Mathematics, Faculty of Agriculture, University of Belgrade, 11000 Belgrade, Serbia; vlaver@agrif.bg.ac.rs

**Keywords:** hydroxyapatite, TiO_2_, nanomaterials, core–shell, antimicrobial

## Abstract

Due to the growing number of people infected with the new coronavirus globally, which weakens immunity, there has been an increase in bacterial infections. Hence, knowledge about simple and low-cost synthesis methods of materials with good structural and antimicrobial properties is of great importance. A material obtained through the combination of a nanoscale hydroxyapatite material (with good biocompatibility) and titanium dioxide (with good degradation properties of organic molecules) can absorb and decompose bacteria. In this investigation, three different synthesis routes used to prepare hydroxyapatite/titanium dioxide nanomaterials are examined. The morphology and semiquantitative chemical composition are characterized by scanning electron microscopy with energy dispersive X-ray analysis (SEM-EDX). The obtained materials’ phase and structural characterization are determined using the X-ray powder diffraction method (XRD). The crystallite sizes of the obtained materials are in the range of 8 nm to 15 nm. Based on XRD peak positions, the hexagonal hydroxyapatite phases are formed in all samples along with TiO_2_ anatase and rutile phases. According to SEM and TEM analyses, the morphology of the prepared samples differs depending on the synthesis route. The EDX analysis confirmed the presence of Ti, Ca, P, and O in the obtained materials. The IR spectroscopy verified the vibration bands characteristic for HAp and titanium. The investigated materials show excellent antimicrobial and photocatalytic properties.

## 1. Introduction

In the last few decades, there has been a lot of bearing interest in developing materials that could partially replace antibiotics. Clinical studies have revealed a growing number of reports related to antibiotic-resistant bacterial pathogens during and after virus infections [1,2]. The pathogenic bacterial infections were Mainly caused by *Staphylococcus aureus*, *Klebsiella pneumonia*, *Escherichia coli*, and *Candida albicans* [1,3,4]. Faced with this dilemma, research has recently focused on new and improved existing materials that have antimicrobial effects and are biocompatible with favorable nanostructured characteristics. Metal oxide compounds have attracted significant attention due to their broad antibacterial activities with favorable photocatalytic performances. Nanoscale materials are widely used because of their advanced properties, originating from a high surface area-to-volume ratio. Antimicrobial nanoparticles have shown excellent and differing activities from their bulk properties [5,6].

Due to unique physiochemical properties having been discovered in the last five decades, metal oxide nanoparticles have been intensively researched, especially materials such as zinc oxide (ZnO), manganese oxide (MgO), titanium dioxide (TiO_2_), and iron oxide (Fe_2_O_3_) [7,8]. One of the oxides that stands out for its performance is TiO_2_, a valuable semiconducting transition metal oxide material. Its unique features, in terms of simple synthesis and economic viability, and, on the other hand, non-toxicity and good resistance to chemical erosion, allow its application as an antibacterial and antifungal agent [9]. Titanium dioxide (TiO_2_) occurs in nature in two allotropic modifications, rutile (s.g., *P4_2_/mnm*) and anatase (s.g., *I4_1_/amd*), with photocatalytic properties which are very important in the degradation of certain organic molecules, but also as a potential antimicrobial material [10]. Additionally, TiO_2_ as a nanopowder is the most frequently used photocatalyst, since it is cheap and very stable with a wide range of applications in pharmacy, medicine, and environment protection [11,12]. Several authors have reported that TiO_2_ nanoparticles are among the most studied nanomaterials due to their photocatalytic antimicrobial activity, exerting excellent bio-related action against bacterial contamination [13,14,15,16]. Using metal oxides in the biomedical field is possible after coating them with a biocompatible material such as hydroxyapatite (HAp). The modification of the photocatalyst can improve the photocatalytic and antimicrobial properties of composite material by inhibiting e–h recombination, which represents the process in which electrons excited from the valence band to the conduction band fall back to the empty state in the valence band, which is also called the hole [17]. By enhancing the charge separation and shifting the wavelength range of absorption, the level of degradation of pollutants and bacteria increases by being adsorbed onto the photocatalyst [18].

Hydroxyapatite (HAp) represents a calcium phosphate material with similar chemical and structural properties to bone tissue. The main advantages of HAp are its bioequivalence, effectiveness, and, most importantly, it does not cause inflammation in the body, so it is considered a very good biocompatible material [19,20]. Since HAp is one of the most researched and tested materials in the field of biomaterials, many years of research have been supported by many papers on its synthesis and methods of obtainment [21,22,23]. Based on previous experience in working with these materials, the method of precipitation from solution gives the best results in regards to obtaining the NP HAp material in combination with low-temperature heating in six hours [24]. This paper presents three simple syntheses of nanostructured hydroxyapatite using acetate precursors instead of nitrate, and the creation of composites with titanium dioxide in the base as well as the use of longer retention times during calcination at relatively low temperatures to obtain nanomaterials with the best structural, morphological, and antimicrobial characteristics. Finally, the main perspective of this paper relies on a simple pathway of the synthesis procedure for obtaining nanostructured core–shell composite materials TiO_2_/HAp with precisely defined structural characteristics, so that they can potentially be utilized as satisfactory antimicrobial materials.

## 2. Results and Discussion

**Figure 1:**
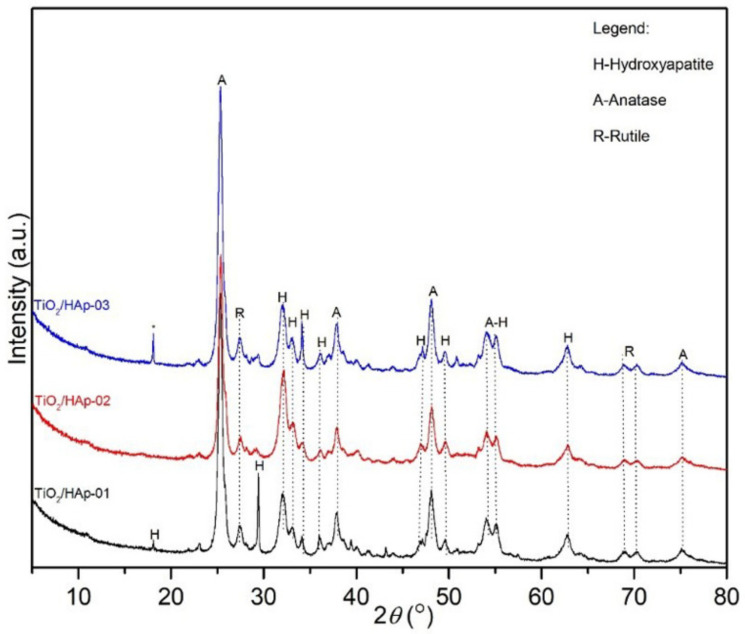
XRD results of TiO_2_/HAp-01 (black line), TiO_2_/HAp-02 (blue line), and TiO_2_/HAp-03 (red line).

The results obtained by the X-ray powder diffraction analysis are given in Figure 1. Results of calculated microstructural parameters are presented in Table 1.

XRD results and identified reflections on the powder diffractograms revealed three phases: anatase, rutile, and hydroxyapatite. The powder diffractogram of the TiO_2_/Hap-01 sample showed an intense, sharp, and clearly defined peak at approximately 30°, which most likely indicated a preferential orientation of stacking hexagonal prismatic nanocrystals of hydroxyapatite, due to the sample preparation. Additionally, the rutile and hydroxyapatite peaks may have overlapped at approximately 30°, which could also contribute to increasing the intensity of the reflection. The presented powder diffractograms showed slightly wider diffraction lines, which indicated very small crystallites with a proper structural order. The thermal treatment also influenced their gradual structural arrangement and oriented growth. The presence of the intense hydroxyapatite peak at approximately 30° was not identified in samples where TiO_2_ was added at the end of the hydroxyapatite precipitation (TiO_2_/HAp-02) nor in the sample synthesized by physically mixing two phases (TiO_2_/HAp-03). Notably, all of the samples had mainly similar phase compositions. The presence of impurities was also identified in the third sample, at about 18° 2*θ*. The microstructural parameters of all samples are presented in Table 1. Based on the phase composition content, the ratio of the phases about 1:1 in the sample TiO_2_/HAp-01 was achieved. This sample had the smallest average Hap and rutile crystallite size, while anatase had the largest average crystallite size. This further indicated that hydroxyapatite was rewarded via TiO_2_ equally during the first synthesis process, so the phases were uniformly deployed. The hydroxyapatite itself had small values of crystallite sizes, indicating nanosized particles. Additionally, the TiO_2_/HAp-01 sample had the highest value of structural strain percentages, meaning the highest number of structural defects, which is desirable for materials with good antimicrobial and photocatalytic properties. For the TiO_2_/HAp-02 and TiO_2_/HAp-03 samples, slightly larger crystallite sizes than the previously mentioned sample were evident, except for a pretty significant deviation in the representation amounts of HAp and TiO_2_ phases. Results showed the highest crystallite size values for hydroxyapatite, while the anatase and rutile phases had similar crystal sizes in all samples. The size of crystallites and microstructural parameters indicated that the synthesis procedure led to the crystal growth, partial arrangement, and absence of an equally distributed volume of initial phases.

**Figure 2:**
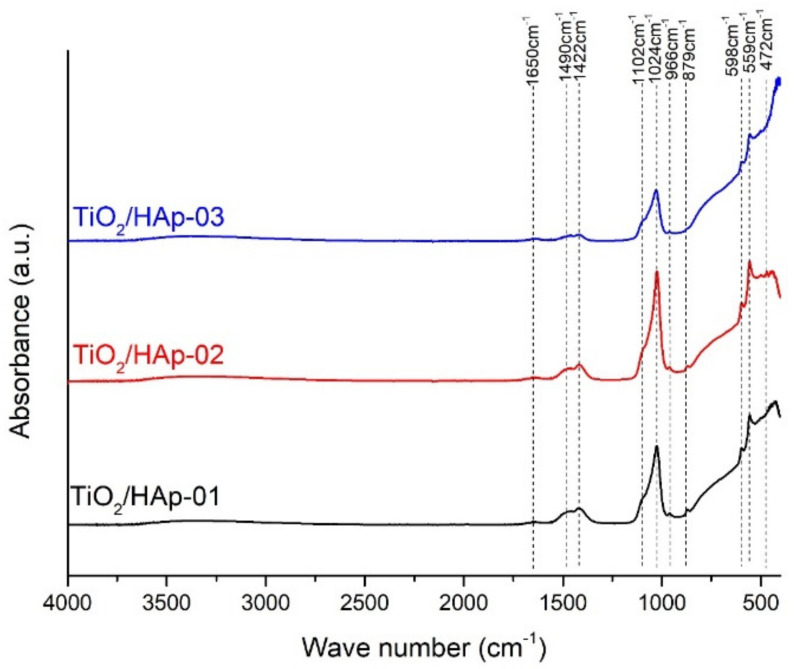
FTIR spectra of TiO_2_/HAp-01, TiO_2_/HAp-02, and TiO_2_/HAp-03.

The FTIR spectra of the composites denoted as TiO_2_/HAp-01, TiO_2_/HAp-02, and TiO_2_/HAp-03 are shown in Figure 2. All detected peaks corresponded to the vibrations of the distinct groups for the TiO_2_ and Hap phases. A general observation indicated that the composite assigned as TiO_2_/HAp-03 had lower intensity peaks. A broad bend between 450 and 800 cm^−1^ originated from the starching vibrations of Ti-O-Ti [25]. Over this broad bend, a few sharp peaks in this region were detected and attributed to specific vibrations of HAp. The dominate bands of PO_4_^3−^, which occurred at 472 cm^−1^, 559 and 598 cm^−1^, 966 cm^−1^, and 1024 and 1102 cm^−1^, corresponded to ν_2_, ν_4,_ ν_1_, and ν_3_, respectively. All band positions agreed with the literature [26,27,28]. Besides modes characteristic for PO_4_^3−^ vibrations in HAp, the bands of CO_3_^2−^ were observed in all spectra. The bands were positioned at 879 and 1490 cm^−1^, ν_2_ and ν_3_, respectively. The presence of these carbonate vibrations indicated the partially carbonated apatite, and it showed that a biological Hap or a B-type of HAp was formed [27]. The carbonate that occurred in samples probably came from the atmosphere. Further, the OH absorption bends at 1650 cm^−1^ (δ(OH)) and low-intensity broad bend at 3520 cm^−1^ (O–H stretching vibrations) suggested that no dehydroxylation had occurred during the formation of the HAp–TiO_2_ nanocomposites [26,29]. The dehydroxylation of HAp took place before the decomposition. During the dehydroxylation, the formation of hydroxyapatite (OHAp) and oxyapatite (OAp) happened, and the decomposition led to tricalcium phosphate (TCP) and tetra calcium phosphate (TTCP) [30]. The dihydroxylation process was avoided by a prolonged thermal treatment at low temperatures.

**Figure 3:**
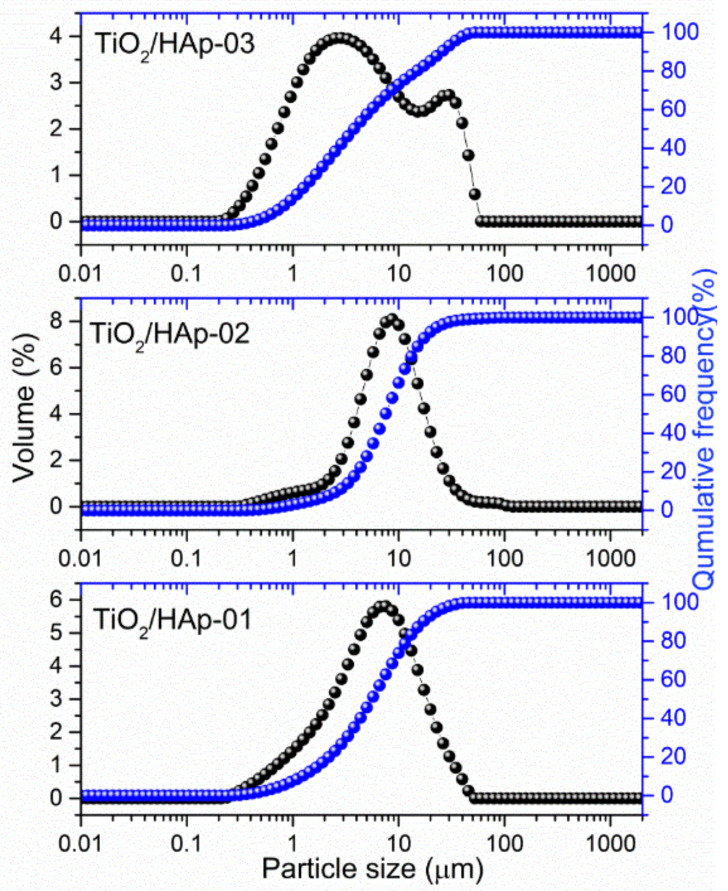
PSD distribution in TiO_2_/Hap-01, TiO_2_/Hap-02, and TiO_2_/HAp-03 samples.

The results of particle sizes and their distributions are shown in Figure 3. Samples TiO_2_/HAp-01 and TiO_2_/HAp-02 showed quite similar distributions, with the presence of fractions with particles between 6 and 8 μm. According to results obtained from TEM images we assumed that these fractions originated from core–shell structured HAp@TiO_2_ composites. The distribution of the TiO_2_/HAp-01 sample shifted slightly lower, indicating thinner and stronger bonded shell particles on the TiO_2_ core. Among this fraction, the presence of a fraction around 1 μm can be noticed, which came from fine, single-phase (HAp or TiO_2_) aggregated particles. Considering that TiO_2_ and the synthesized HAp originated from the nanosized powder, it is impossible to say with certainty from whom. Contrary to the mentioned samples, the particle size distribution for TiO_2_/HAp-03 showed a significantly different appearance. Namely, a bimodal distribution could be detected, with the majority of particles in the range of 2–3 μm, and the presence of large agglomerates bigger than 12 μm.

A measured value of the *plan range*, which was below three for TiO_2_/HAp-01 and TiO_2_/HAp-02, indicated a relatively narrow distribution. The third sample’s span was much higher, pointing to a broader distribution.

**Figure 4:**
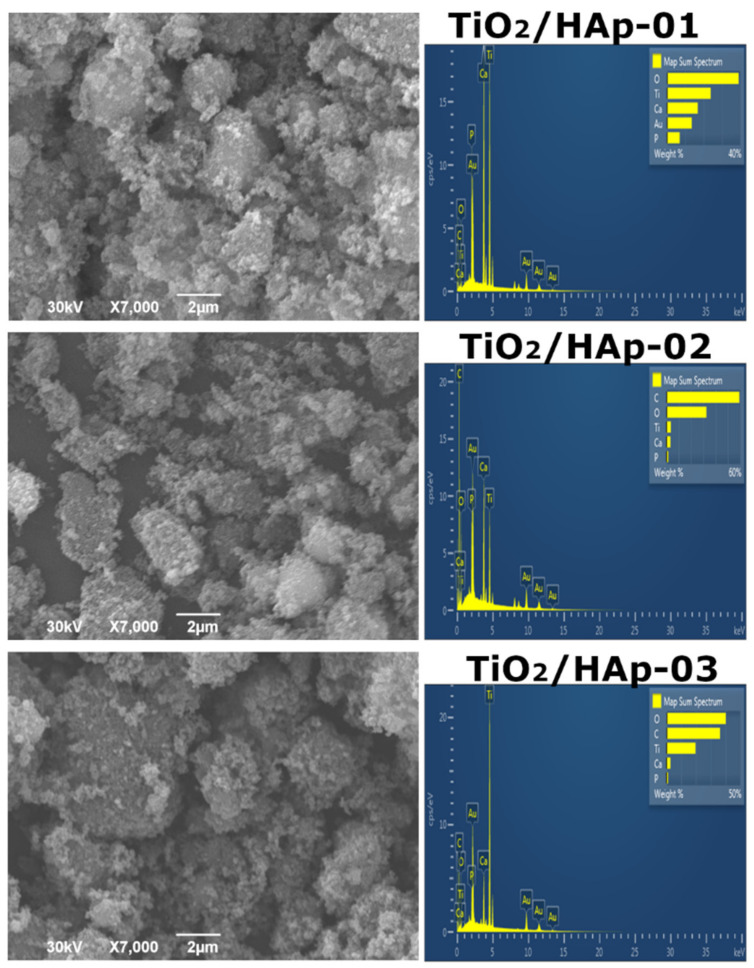
SEM results with inserted EDS spectra TiO_2_/HAp-01, TiO_2_/HAp-02, and TiO_2_/HAp-03.

The morphology of the synthesized samples was investigated by a SEM-EDS analysis, and obtained results were presented in Figure 4. The results for the first sample, TiO_2_/HAp-01 (Figure 4), indicated the uniform accumulation of HAp particles over TiO_2_. Larger TiO_2_ agglomerates were 1.5–2 μm in size; smaller HAp particles were below 100 nm. Figure 4 represents the TiO_2_/Hap-02 sample results, which evidently showed that the sample was homogeneous, but this type of synthesis did not form the core–shell structure. It can be stated that good composite material was developed. The existence of separate HAp and TiO_2_ phases is evident. The TiO_2_/HAp-03 sample (Figure 4) indicated the presence of well-mixed phases, but again, no core–shell structure was formed due to the synthesis method. Based on the EDS spectra, the Ca/P ratio was between 1.60 and 1.73 for all samples. For the TiO_2_/HAp-01 sample, the ratio of Ca/P was calculated from EDS to obtain a value of approximately 1.67, which corresponded to the theoretical value characteristic of stoichiometric hydroxyapatite [31].

**Figure 5:**
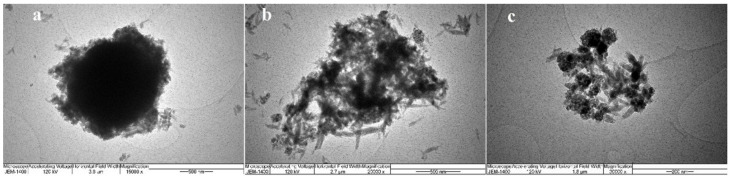
TEM images of (**a**) TiO_2_/HAp-01, (**b**) TiO_2_/HAp-02, and (**c**) TiO_2_/HAp-03.

Considering that the SEM images indicated the nanodimensions of the obtained powders, a further investigation of the morphology was needed. The TEM images of all prepared samples were taken in that respect, and the obtained results were presented in Figure 5.

The TiO_2_/HAp-01 sample implied a core–shell structure. The prismatic hexagonal HAp crystals were evenly distributed around the TiO_2_ crystal; thus, creating the core and shell structure. It can be seen from the figure that the shell consisted of nanohydroxyapatite crystal grains of similar thickness in all parts. The average grain size of HAp was approximately 20 nm. Considering the calculated crystallite size values for this sample by XRD analysis, two crystallites from one crystal grain of hydroxyapatite were obtained. Figure 5b also revealed the nanocrystal morphology of the TiO_2_/HAp-02 and TiO_2_/HAp-03 samples with randomly oriented TiO_2_ and HAp nanocrystalline grains. The absence of a core–shell structural formation in these two samples was evident.

**Figure 6:**
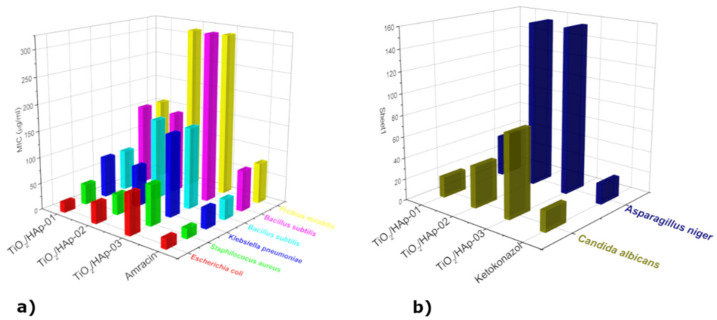
(**a**) MIC properties of TiO_2_/HAp-01, TiO_2_/HAp-02, and TiO_2_/HAp-03 in relation to Amracin; (**b**) MIC properties of TiO_2_/HAp-01, TiO_2_/HAp-02, and TiO_2_/HAp-03 materials in relation to Ketoconazole.

Minimum inhibitory concentrations were determined for eight selected indicator strains, presented in the form of histograms in Figure 6. The results presented revealed the antimicrobial activity of samples within the concentration range of 19.53 μg/mL to 312.50 μg/mL. The highest susceptibility to sample TiO_2_/HAp-01 among the bacteria tested was exhibited by *E. coli* ATCC 25922 (MIC = 19.53 μg/mL), followed by strains of *S. aureus* ATCC 25923 (MIC = 39.1 μg/mL), *B. subtilis* ATCC 6633, *K. pneumonia* ATCC 13883 (MIC = 78.125 μg/mL), *P. mirabilis* ATCC 14153, and *P. Vulgaris* ATCC 13315 (MIC = 156.25 μg/mL). Among the fungi, *C. Albicans* ATCC 10231 (MIC = 19.53 μg/mL) showed the highest susceptibility, and *A. niger* ATCC 16404 (MIC = 39.1 μg/mL) the lowest (Figure 6b). This sample had a strong antimicrobial potency compared to a standard antibiotic/antifungal. The results obtained by the antimicrobial activity assay confirmed that the TiO_2_/HAp-02 sample possessed a strong to moderately strong antimicrobial potency relative to the standard antibiotic/antifungal. The most sensitive bacteria to the TiO_2_/HAp-02 sample was E. coli ATCC 25922 and *S. aureus* ATCC 25923 (MIC = 39.1 μg/mL), followed by *K. pneumoniae* ATCC 13883 (MIC = 78.125 μg/mL). *P. Vulgaris* ATCC 13315 and *B. subtilis* ATCC 6633 showed a moderately strong antimicrobial potency (MIC = 156.25 μg/mL), while *P. mirabilis* ATCC 14153 showed a moderate antimicrobial potency (MIC = 312.5 μg/mL).

Among the fungi, *C. Albicans* ATCC 10231 (MIC = 39.1 μg/mL) showed the highest sensitivity, and *A. niger* ATCC 16404 (MIC = 156.25 μg/mL) the lowest. The TiO_2_/HAp-03 sample showed the weakest antimicrobial potency on all strains tested compared to a standard antibiotic or antifungal. The most sensitive bacteria to the TiO_2_/HAp-03 sample were *E. coli* ATCC 25922 and *S. aureus* ATCC 25923, and from fungus *C. Albicans* ATCC 10231 (MIC = 78.125 μg/mL). All other tested strains showed a moderate antimicrobial potency compared to the standard ones, with values ranging from 156.25 to 312.5 μg/mL. The TiO_2_/HAp-01 sample showed very strong antimicrobial activity. In this sample, the most sensitive bacterium was *E. coli* inches and the fungus *C. Albicans*, which had the same minimum inhibitory concentration value as a standard antibiotic or antifungal. It can be assumed that this type of core–shell structured nanocomposite material led to a combined effect, where the bacteria was attracted to the hydroxyapatite nanolayer, and further by the photocatalytic effect, it was decomposed by titanium dioxide, which led to bacteria death [32,33]. The connection between bacteria and HAp occurred due to the formation of hydrogen and covalent bonds among the carbohydrate groups, in the bacteria’s membrane, and hydroxyl and phosphate groups of HAp [27].

The possible reason for the antimicrobial action of the composite was the cell wall and membrane damage. Once it entered a cell, TiO_2_-based photocatalysts produced highly oxidizing free radicals, which enabled the translation of genetic information and the synthesis of proteins, destroying bacteria. In terms of the excellent antifungal properties, a similar mechanism could be assumed. The composite particles were linked with the ergosterol molecules, the gradient molecules of the fungal membrane. In such contact, damages of the membrane are highly possible, initiating the fungal cell’s destruction [34].

In general, upon the illumination of the photocatalyst with an adequate wavelength light, an electron was transferred from the valence band (VB) to the conduction band (CB). In this process, an electron–hole pair was formed. Separated charges could include various reactive oxygen species (ROS) in aqueous solution, such as hydroxyl radical (O•H), hydroperoxyl radical $\left({\mathrm{HO}}_{2}^{•}\right)$, hydroperoxide ($H_{2}O_{2}$), and superoxide ions from oxygen in the air ($O_{2}^{•-}$) that could oxidize organic materials and destroy bacteria [28,34].

We assumed that the TiO_2_/HAp-01 sample showed very strong antimicrobial activity because it had a core–shell structure. This structure broadly inhibited the growth and development of microorganisms. Literature data showed that defects in the crystal lattice can be placed where separated charge carriers can be trapped. In this way, the fast recombination of the charge carriers can be suspended [35,36,37,38]. As we explained earlier in the XRD analysis, the TiO_2_/HAp-01 sample showed the highest amount of crystal lattice defects and the preferential orientation of HAp crystals, which could be the reason for the highest achieved antimicrobial behavior among the three synthesized powders. Additionally, it was shown that the gradual structural arrangement and oriented growth during subsequent thermal treatment did not have a detrimental influence on particle size. The powder kept the nanosized grains, which positively affected the photocatalytic and antimicrobial properties [34]. Further, the optimal ratio of the rutile/anatase phase for the enhanced antimicrobial activity was about 1:1, which was achieved only in the TiO_2_/HAp-01 sample.

Some authors have suggested that HAp also acts as a source of oxidative spaces [39,40,41]. They showed that upon UV irradiation, the PO_4_ groups changed due to the formation of oxygen vacancies on the surfaces. The oxygen from the air was further activated due to the electron trapped on the vacancy in the HAp, followed by the formation of the unstable $O_{2}^{•-}$ species. Therefore, the synergetic influence of a few factors was responsible for this particular sample’s improved antimicrobial and photocatalytic properties.

Samples labeled as TiO_2_/HAp-02 and TiO_2_/HAp-03 showed moderate or low antimicrobial action for most of the tested microbial, compared to the used antibiotic and antifungal. The lower activity could be explained by the higher crystallinity, the disturbed optimal ratio of the anatase/rutile phases, and a lower level of crystal disorder. In addition, the microstructure of these samples was significantly different; there was no strong connection between phases, which would influence the coupling and disruption of the ordered crystal structure of composite phases.

**Figure 7:**
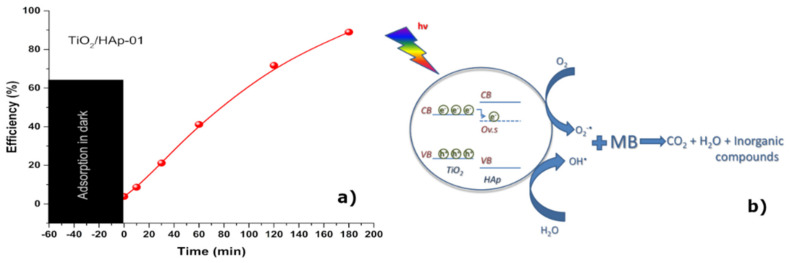
(**a**) Photocatalytic efficiency of the TiO_2_/HAp-01; (**b**) possible mechanism of the MB photocatalytic degradation in the presence of TiO_2_/HAp-01.

To confirm the photocatalytic behavior of the sample TiO_2_/HAp-01, which showed the best antimicrobial properties, the degradation of MB in the presence of a synthesized photocatalyst was recorded. Figure 7 reveals the photocatalytic efficiency of the TiO_2_/HAp-01 sample under UV–Vis illumination. The TiO_2_/HAp-01 sample showed a near 90% efficiency after 3 h of illumination.

The MB or any other organic compound would photodegrade by the following reaction (1):

MB + Reactive radicals → Intermediates + Reactive radicals → CO_2_ + H_2_O + HA (1)

where HA indicates the appropriate inorganic acid [42,43]. The formation of the reactive radicals occurred via the following reactions [27]:

(2) $${\mathrm{TiO}}_{2}+h\nu \;\to \;e_{\mathrm{CB}}^{-}+{\;h}_{\mathrm{VB}}^{+}$$

(3) $$e_{\mathrm{CB}}^{-}+O_{2}\;\to \;O_{2}^{•-}$$

(4) $$O_{2}^{•-}+H^{+}\;\to \;{\mathrm{HO}}_{2}^{\cdot }$$

(5) $${\mathrm{HO}}_{2}^{•}+H^{+}+O_{2}^{•-}\;\to \;H_{2}O_{2}^{}+O_{2}$$

(6) $$H_{2}O_{2}+e_{\mathrm{CB}}^{-}\;\to \;{\mathrm{OH}}_{}^{-}+{\mathrm{OH}}^{\cdot }$$

The main disadvantage of the TiO_2_ catalyst is its fast recombination of the photo-generated electron–hole pairs [42]. Such a problem could be overcome by increasing the number of defects, doping titania, or synthesizing the composite material and the formation of a heterojunction. With the latest method, the photo-generated electrons from the CB of TiO_2_ could be transferred to the oxygen defect level (Ov.s) from the electron state change of -PO_4_^3−^ in HAp and, thus, the prolonged lifetime of e^−^. In such a case, the recombination would be suspended and the reaction described by Equations (1)–(6) could continue [44]. Keeping in mind that the most intense contacts between compounds were established within TiO_2_/HAp-01, along with the increased amount of surface defects, we could conclude that this scenario was the most probable for the investigated sample. The mechanism of the photocatalytic degradation of MB in the presence of the TiO_2_/HAp-01 is presented in Figure 7b.

## 3. Materials and Methods

For the synthesis of the composite material’s TiO_2_/HAp nanomaterial, the following reagents were used: commercially available titanium (IV) oxide (99.5%, p.a. Sigma Aldrich, St. Louis, MO, USA, nanostructured rutile/anatase). Solution precipitation method for hydroxyapatite synthesis was used by titration of the solution of 0.5 M Ca(OH)_2_ (p.a. ≥96%, Carlo Erba) with 0.3 M NaH_2_PO_4_ + H_2_O (≥99.0%, Sigma Aldrich) at a temperature of approximately 80 °C, and 5 mL of NH_4_OH was used for pH adjustment (25% solution, Fisher Chemical, Waltham, MA, USA). The obtained powder was washed five times in distilled water and once in ethanol, and allowed to dry for 24 h at 60 °C. Here, three simple synthesis pathways were performed to prepare TiO_2_/Hap composites. For a sample denoted as TiO_2_/HAp01, TiO_2_ powder was added to a beaker with Ca(OH)_2_ solution at the beginning of titration with NaH_2_PO_4_. The sample TiO_2_/Hap02 was synthesized by adding TiO_2_ at the end of the titration process. The third synthesis, TiO_2_/HAp03, involved a physical mixing of TiO_2_ and synthesized HAp powders. All syntheses were performed in a TiO_2_:HAp = 1:1 phase ratio. After powder preparation, all powders were calcined in an air atmosphere at 300 °C for 6 h.

Phase and microstructural analyses of all samples were determined using the X-ray powder diffraction (XRD) method using Rigaku, Ultima IV diffractometer with CuKα1,2 tube, equipped with D/TeX ultra-high-speed detector. The generator voltage (40.0 kV) and a current (40.0 mA) were used for all powders in a range from 5 to 80° 2θ, with a scanning step size of 0.02° and a scan rate of 5°/min. Si-monocrystalline sample carrier was used for sample preparation. The PDXL2 (Ver. 2.8.4.0) software, Rigaku, Tokyo, Japan was used to evaluate the phase identification and refine the material’s microstructure properties; the crystallite sizes and lattice strain were determined by the Williamson–Hall method. Quantitative phase composition was determined using the RIR method [45]. All obtained powders were identified using the ICDD database [46], the selected card was used for phase identification: anatase (PDF: 01-089-4921), rutile (PDF: 01-072-4819), and hydroxyapatite (PDF: 01-089-4405).

Fourier transform infrared (FT-IR) spectra were recorded on a ThermoScientifc™ Nicolet™ iS™10 FT-IR spectrometer equipped with attenuated total reflectance (ATR) accessory. The ATR/FT-IR measurements were conducted in the wavenumber region of 400–4000 cm^−1^, with a resolution of 4 cm^−1^. Spectra were recorded at room temperature.

The laser diffraction on Mastersizer 2000 Malvern Instruments Ltd. (Malvern, UK) investigated the particle size distributions, where powders were dispersed in distilled water. The used instrument covered the particle size range of 0.02–2000 μm. Prior to measurements, the powders were subjected to low-power ultrasound treatment for 5 min.

SEM-EDS analysis was performed on a scanning electron microscope JEOL-JSM 6390 LV, JEOL Ltd., Tokyo, Japan. The samples were previously Ag-coated before analysis.

TEM analysis was performed on JEM-1400. Samples were prepared by sonication of 1.7 mg of the powder in methanol for 45 min, after which one drop was deposited onto the carbon mesh.

For the potential application of these materials, antimicrobial measurements were performed. The antimicrobial activity of the samples was tested in vitro against the following bacteria: Staphylococcus aureus ATCC 25923, Klebsiella pneumonia ATCC 13883, Escherichia coli ATCC 25922, Proteus Vulgaris ATCC 13315, Proteus mirabilis ATCC 14153, and Bacillus subtilis ATCC 6633; it was also tested against the following fungi: Candida albicans ATCC 10231 and Aspergillus niger ATCC 16404. The fungi were reseeded on potato-glucose agar, on which they developed for seven days at a room temperature of 20 °C under alternating day–night light conditions. They were reseeded on a new potato-glucose substrate, on which they sat for another seven days. The reseeding procedure was performed four times, and after the process, the pure cultures needed for determination were obtained. The Laboratory of Mycology, Department of Microbiology, Institute Torlak, Belgrade, Serbia, confirmed the identification of the microorganism tests. The samples’ minimal inhibitory concentrations (MIC) against tested bacteria were determined using a microdilution method in 96-well microliter plates [47]. All tests were performed in Muller–Hinton broth (MHB), except for the yeast when Sabouraud dextrose broth was used. A volume of 100 μL stock solutions of samples (in 10 % DMSO, 2 mg/mL) was pipetted into the first row of the plate. The rest of the wells 50μL, supplemented with Tween 80 at a final concentration of 0.5% (*v*/*v*) for sample analysis, were added. A volume of 50 μL from the first test well was pipetted into the second well of each microliter line, and then 50 μL of scalar dilution was transferred from the 2nd to the 12th well. To each well, 10 μL of resazurin indicator solution (prepared by dissolving a 270 mg tablet in 40 mL of sterile distilled water) and 30 μL of nutrient broth were added. The final procedure involved 10 μL of bacterial suspension (106 CFU/mL) and yeast spore suspension (3 × 10^4^ CFU/mL) addition to each well. The growth conditions and the sterility of the medium for each strain were carefully checked. One of the most standard and frequently used antibiotics, Amracin, was used to control the sensitivity of the tested bacteria, and Ketoconazole, on the other hand, was used as a control against the tested yeast. The final preparation involved wrapping plates loosely with cling film to ensure that bacteria did not become dehydrated and prepared them in triplicate. After that, they were placed in a regime to the incubator at 37 °C for 24 h for the bacteria and at 28 °C for 48 h for the yeast. Monitoring of color changes was performed visually. Any change in color from colorless or purple to pink was considered positive. The MIC value was taken as the lowest concentration at which color change occurred. Average data obtained from three values were calculated, and those were the MIC for the tested compounds and standard drug.

A photocatalytic activity test was performed for the selected TiO_2_/Hap-01 sample. Solution of 10 ppm of methylene blue (MB) day was prepared by dissolving MB powder in appropriate distilled water volume. To separate the degradation process from adsorption, it was magnetically stirred for 1 h in the dark before suspension exposure to illumination to reach the adsorption–desorption equilibrium. After one hour in the dark, the concentration of MB was measured, and this measured value was labeled as Co. During illumination, the suspension was continuously stirred with a magnetic stirrer. Defined time ranges were used for 3 mL aliquots by a centrifuge (8000 rpm for 10 min) to release particulate matter from the solution before absorbance measurements. Concentrations of MB in solution were measured by a BC Cintra UV–Vis spectrophotometer in the wavelength range of 450–750 nm, and the concentration of MB was calculated according to the absorbance value at 665 nm.

## 4. Conclusions

Based on the obtained results, it was concluded that the method of precipitation of hydroxyapatite where titanium dioxide was added at the very beginning of the precipitation reaction led to the formation of core–shell structural nanocomposite materials. The XRD results revealed that the obtained materials had a proper structural arrangement. In contrast, the smallest crystallite sizes and higher structural strains had the TiO_2_/Hap-01 material, confirming the core–shell structure. The FTIR analysis confirmed the main vibration bands of HAp and TiO_2_, and also confirmed that the high-temperature calcium phosphate phases were not formed. Both methods revealed that calcination at 300 °C for a longer retention time led to the proper structural arrangement of synthesized nano-TiO_2_/HAp, without a second phase or high-temperature phosphate phase formation. Additionally, the desired ratio of anatase and rutile phases was reviled for this sample, which was responsible for the excellent photocatalytic effect. The TiO_2_/Hap-01 sample had a narrow particle size distribution, but was somewhat more comprehensive than TiO_2_/Hap-02, indicating the attachment of thin bonded shell particles of HAp onto the TiO_2_ core. For the third sample, the distribution was quite bimodal and uneven. SEM-EDS results confirmed the previously obtained results, indicating the best covering of TiO_2_ nanoparticles by accumulating HAp particles over them. It was revealed by the EDS analysis that smaller HAp particles almost thoroughly coated the TiO_2_ particles. Larger TiO_2_ agglomerates were 1.5–2 μm in size; smaller HAp particles were below 100 nm. The other two samples revealed an uneven covering of TiO_2_ by HAp. The TEM results confirmed the core–shell TiO_2_/Hap-01 nanocomposite material, with the inner TiO_2_ grain covered by prismatic hexagonal hydroxyapatite crystal grains. The material with the best achieved structure and morphology, TiO_2_/Hap-01, was examined for photocatalytic purposes. The MB degradation of nearly 90% was achieved after 3 h of illumination, revealing a highly efficient photocatalytic material. Antimicrobial measurements were conducted in the meaning of potential functional applications of this material, such as skin coatings or partially antibiotic substituent due to pathogen infection or fungal infection. The obtained TiO_2_/Hap-01 sample showed very strong antimicrobial activity. In this sample, the most sensitive bacterium was E. coli and the most sensitive fungus was C. Albicans, both of which had the same minimum inhibitory concentration value as a standard antibiotic or antifungal, respectively. We assumed that the TiO_2_/HAp-01 sample showed very strong antimicrobial activity because it had a core–shell structure, appropriate amounts of defects, and an anatase/rutile ratio. This structure broadly inhibited the growth and development of microorganisms.

## Figures and Tables

**Table 1:** Microstructural parameters of TiO_2_/HAp-01, TiO_2_/HAp-02, and TiO_2_/HAp-03.

Sample	Phase	Crystallite Size (Å)	Strain (%)	Phase Content (%)
TiO_2_/HAp-01	HAp	81.20 (4)	0.45 (8)	43.5 (6)
	Anatase	186.00 (9)	0.56 (5)	28.5 (9)
	Rutile	24.59 (2)	0.79 (3)	28.0 (6)
TiO_2_/HAp-02	HAp	87.20 (3)	0.43 (6)	61.2 (6)
	Anatase	113.70 (4)	0.14 (9)	30.3 (4)
	Rutile	70.9 (3)	0.78 (4)	8.5 (4)
TiO_2_/HAp-03	HAp	130.6 (5)	0.42 (6)	81.3 (7)
	Anatase	134.7 (5)	0.39 (6)	16.7 (7)
	Rutile	117.3 (6)	0.09 (5)	2.0 (7)

## Data Availability

Not applicable.
